# Supplementary material for: Spatial distribution of core monomers in acrylamide-based core-shell microgels with linear swelling behaviour
Source: Sci Rep. 2019 Sep 25;9:13812. doi: 10.1038/s41598-019-50164-6 (PMC6761195; doi:10.1038/s41598-019-50164-6)
Supplement: Supplementary file 1 — Supplementary Information [file 41598_2019_50164_MOESM1_ESM.pdf]

Supplementary Information

Spatial distribution of core monomers in acrylamide-based core-shell microgels with linear swelling behaviour

Marian Cors[1, 2], Oliver Wrede[1], Lars Wiehemeier[1], Artem Feoktystov[3], Fabrice Cousin[4], Thomas Hellweg[1,5*], Julian Oberdisse[2*]

[1] *Department of Physical and Biophysical Chemistry, Bielefeld University, Universitätsstr. 25, 33615 Bielefeld, Germany*
[2] *Laboratoire Charles Coulomb (L2C), University of Montpellier, CNRS, 34095 Montpellier, France.*
[3] *Forschungszentrum Jülich GmbH, Jülich Centre for Neutron Science JCNS at Heinz Maier-Leibnitz Zentrum MLZ, 85748 Garching, Germany.*
[4] *Laboratoire Léon Brillouin, UMR 12 CEA/CNRS, CEA Saclay, 91191 Gif Sur Yvette, France*
[5] *Lund Institute of Advanced Neutron and X-ray Science (LINXS), IDEON Building: Delta 5, Scheelevägen 19, 22370, Lund, Sweden.*

* *Authors for correspondence : thomas.hellweg@uni-bielefeld.de, julian.oberdisse@umontpellier.fr*

Contrast variation

Two pNNPAM microgel systems (CCC = 1.9 mol%, same as for the shell) have been purpose-synthesized for contrast variation using H_2O/D_2O. The scattering length density (SLD) of the effective partially deuterated D7-pNNPAM microgel including the cross-linker was determined to be $5.54 \cdot 10^{10}$ cm^{-2} (see Figure S1), resulting in a density of 1.24 g cm^{-1} based on the theoretical value of $4.47 \cdot 10^{10}$ cm^{-2} at a density of 1.00 g cm^{-1}. The contrast variation of H-pNNPAM revealed a SLD of $1.05 \cdot 10^{10}$ cm^{-2} and is displayed in Figure S2.

The *q*-values of the contrast variation of D7-pNNPAM in Figure S1a are from black squares to brown right facing triangles: (0.005, 0.00625, 0.00816, 0.01005, 0.01192, 0.0138, 0.0157) $Å^{-1}$ and the *q*-values of H-pNNPAM in Figure S2a are (0.00436, 0.00624, 0.00814, 0.01002, 0.01189, 0.01377, 0.01567) $Å^{-1}$. They are chosen to be all in the low-*q* range. The identical match-point in Figure S1a and S2a for different *q*-values can be seen as an extrapolation (of a constant) to $q \to 0$ as done in classical contrast variation. The superposition in Figure S1c and S2c indicates that the polymer chains are the only source of contrast in these experiments, and the contrast variation gives identical results on all measurable scales.

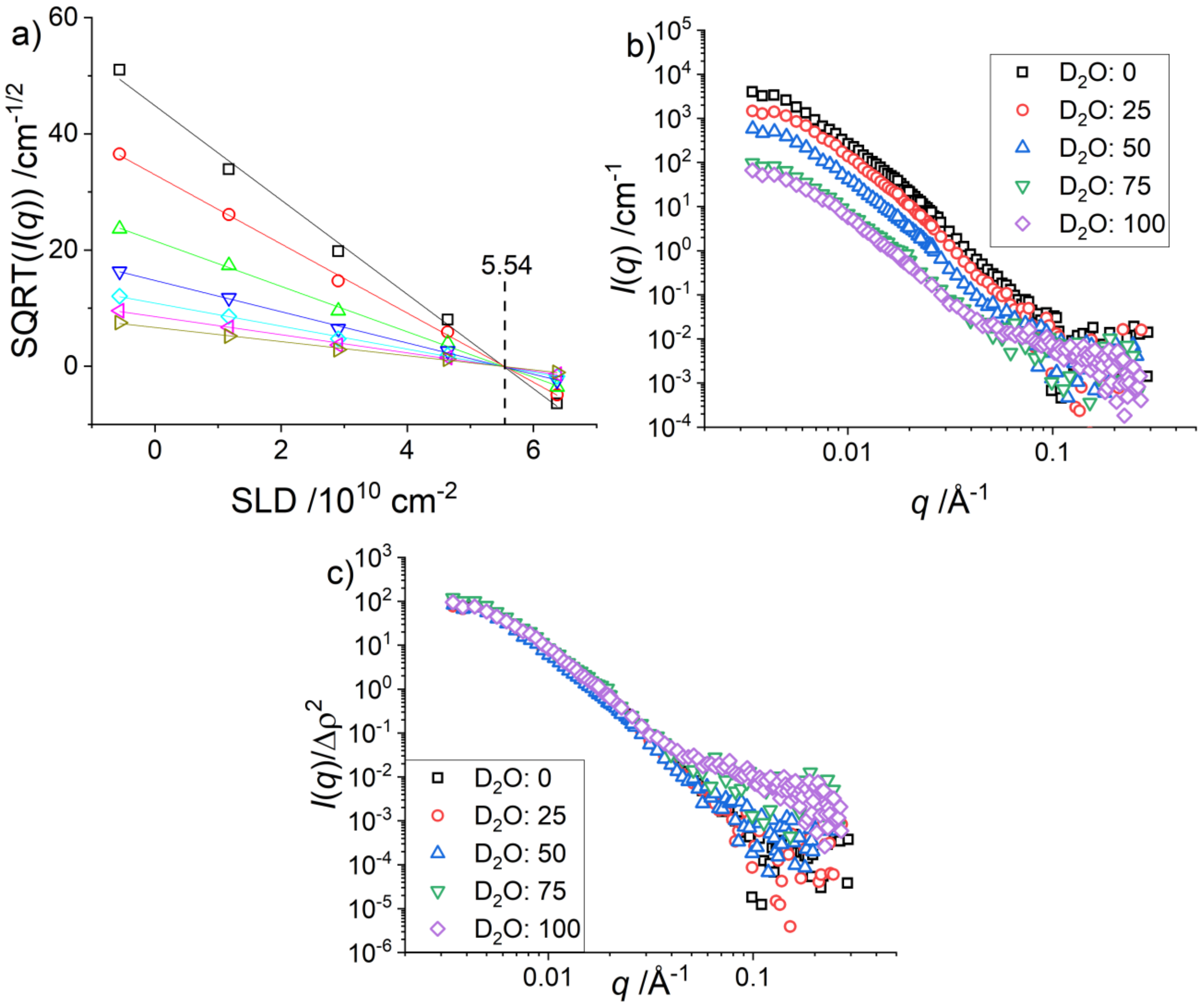

Figure S1: Contrast variation of D7-pNNPAM microgels with a CCC of 10 mol%. **(a)** Plotted is the sign corrected square root of the coherent intensities vs. the SLD of the solvent varied from pure H_2O to pure D_2O (-0.561, 1.173, 2.907, 4.641, 6.375) $\cdot$ 10^{10} cm^{-2} for seven q-values ((0.005, 0.00625, 0.00816, 0.01005, 0.01192, 0.0138, 0.0157) $\cdot$ $Å^{-1}$). The dashed line indicates the match point of 5.54 $\cdot$ 10^{10} cm^{-2}. **(b)** Scattering functions from the contrast variation. **(c)** Contrast scaled scattering functions.

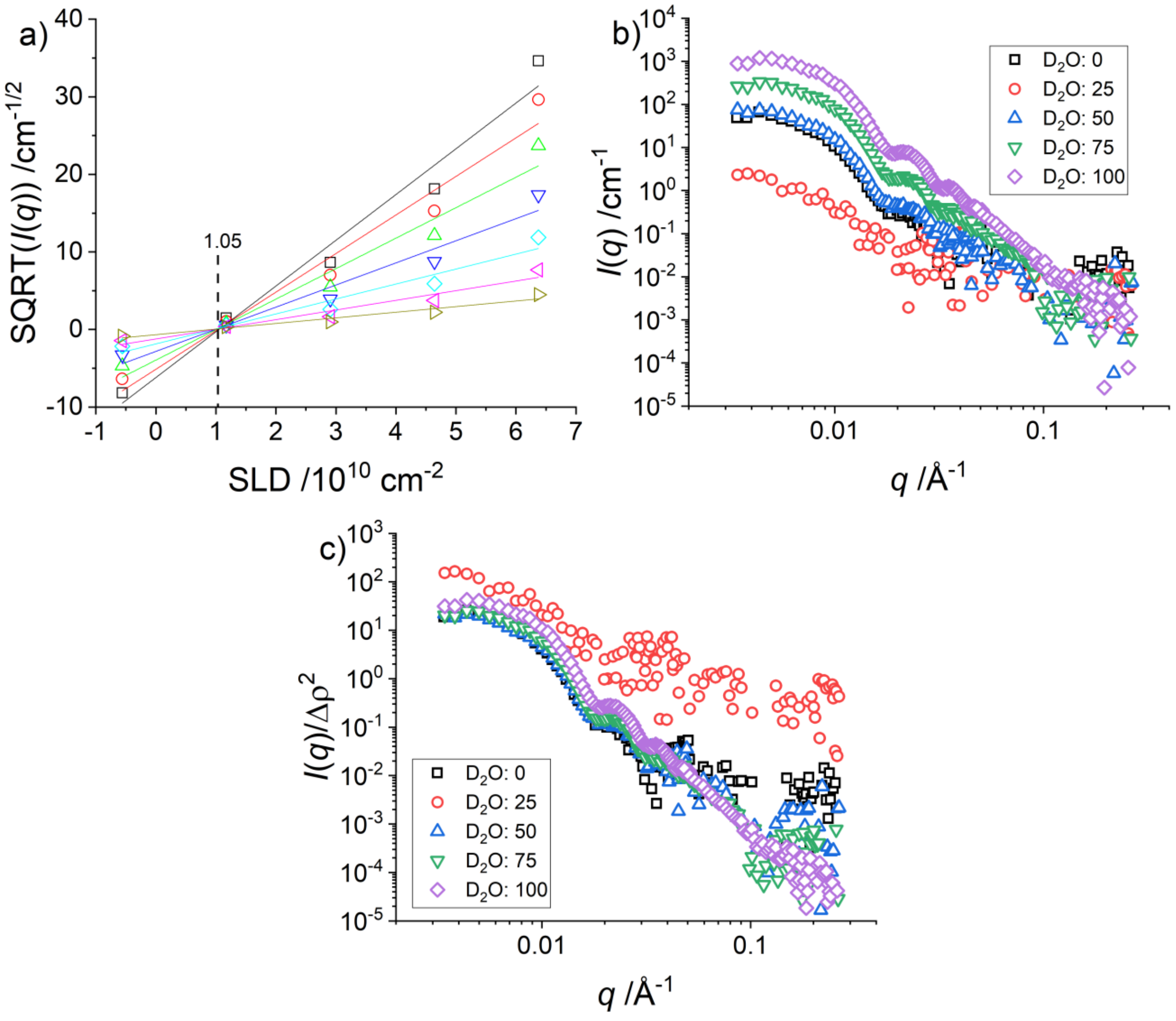

Figure S2: Contrast variation of H-pNNPAM microgels with a CCC of 10 mol%. **(a)** Sign-corrected square root of the coherent intensities vs. the SLD of the solvent varied from pure H_2O to pure D_2O (-0.561, 1.173, 2.907, 4.641, 6.375) · 10^{10} cm^{-2} for seven q-values ((0.00436, 0.00624, 0.00814, 0.01002, 0.01189, 0.01377, 0.01567) · $Å^{-1}$). The dashed line indicates the match point of 1.05 · 10^{10} cm^{-2}. **(b)** Scattering functions from the contrast variation. **(c)** Contrast-scaled scattering functions.

Effect of the CCC on pNIPMAM-pNNPAM microgels

The effect of the CCC on the density profiles of H-pNIPMAM-H-pNNPAM microgels at 55 °C is discussed in the main article. Here, we present the experimental SANS data (Figure S3a and S4a) and the density profiles (Figure S3b and S4b), respectively.

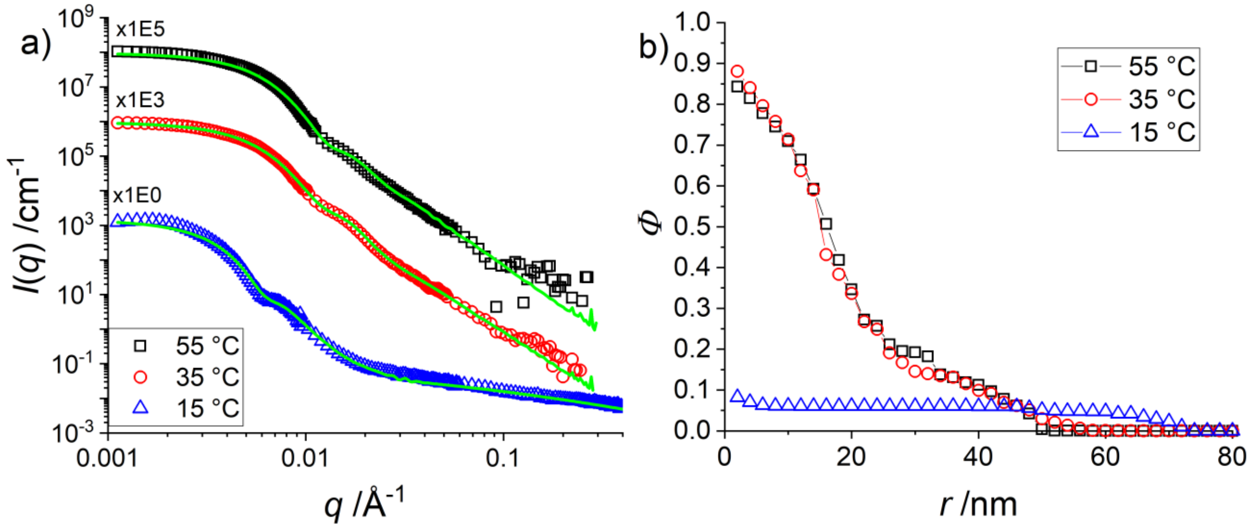

Figure S3: H-pNIPMAM core with H-pNNPAM shell with 5 mol% CCC at different temperatures. **(a)** intensity shifted scattering curves from SANS experiments. **(b)** Revealed monomer density profiles by a RMC analysis.

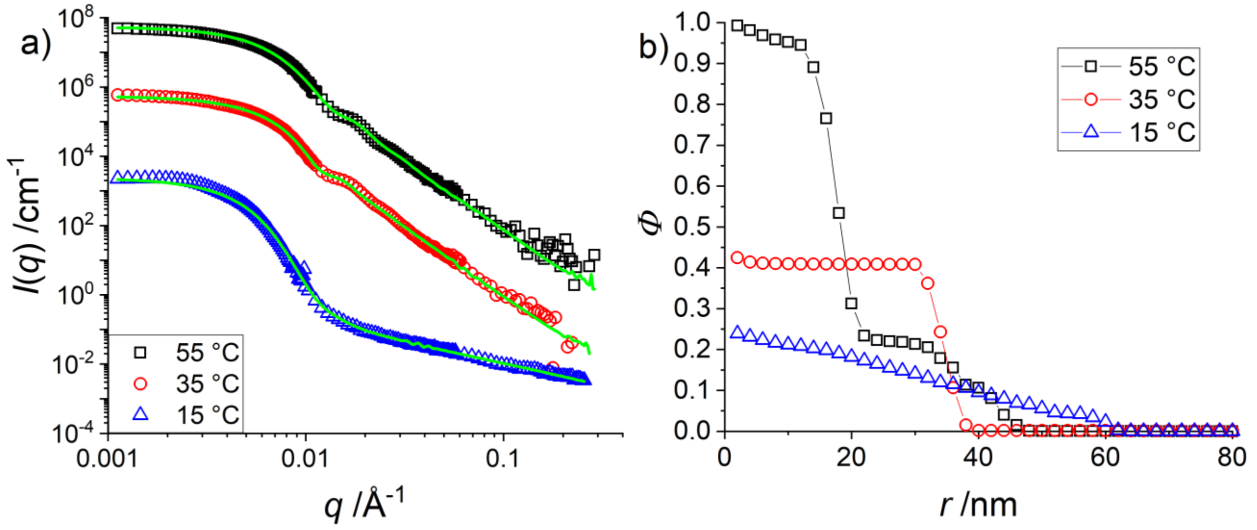

Figure S4: H-pNIPMAM core with H-pNNPAM shell with 15 mol% CCC at different temperatures. **(a)** intensity shifted scattering curves from SANS experiments. **(b)** Revealed monomer density profiles by a RMC analysis.

Particle mass determination by analysing the structure factor peak

Calculating the number of monomers in one microgel by analysing $I(q\to 0)$ reveals a N_{mono} of 160 000 monomers, if monodispersity is assumed.

As a second method, the number of monomers was calculated from the position of the first structure factor peak. It is found that this leads to a number of pNIPMAM-core monomers N_{mono} which is of the same order of magnitude as the one determined from $I(q\to 0)$ (factor 1.2).

T / °C	q_{max} / Å^{-1}	N_{mono} (monodisperse)
15	0.00295	198 000
30	0.00285	219 000
35	0.00297	194 000
40	0.00301	186 000
55	0.00299	190 000
Average	0.002954	197 000

Table S1: Calculated numbers of monomers of a pNIPMAM microgel from the first structure factor oscillation at different temperatures.

Comparison of a pNIPMAM core with and without shell

Figure S5 shows the monomer density profiles of H-pNIPMAM cores without shell and with a contrast matched D7-pNNPAM Shell with 5, 10 and 15 mol% CCC at 55 °C.

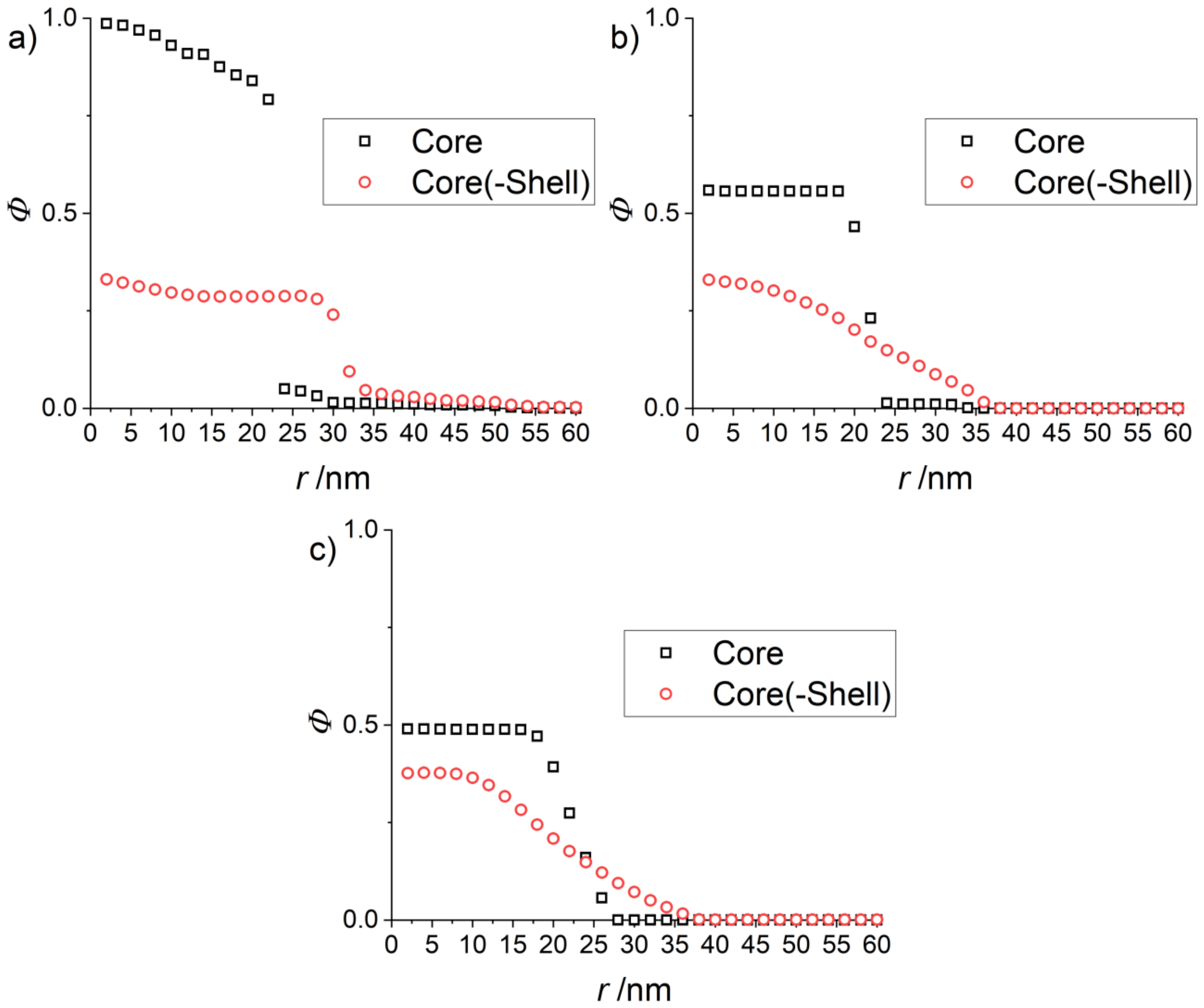

Figure S5: Density profiles of H-pNIPMAM cores without and with contrast matched D7-pNNPAM shell at 55 °C **(a)** CCC = 5 mol% **(b)** CCC = 10 mol% **(c)**CCC = 15 mol% **.**

Figure S6 shows the dependence of the temperature (15°C, 35°C, and 55°C) of a H-pNIPMAM core without shell and with a contrast matched D7-pNNPAM Shell with a CCC of 10 mol%.

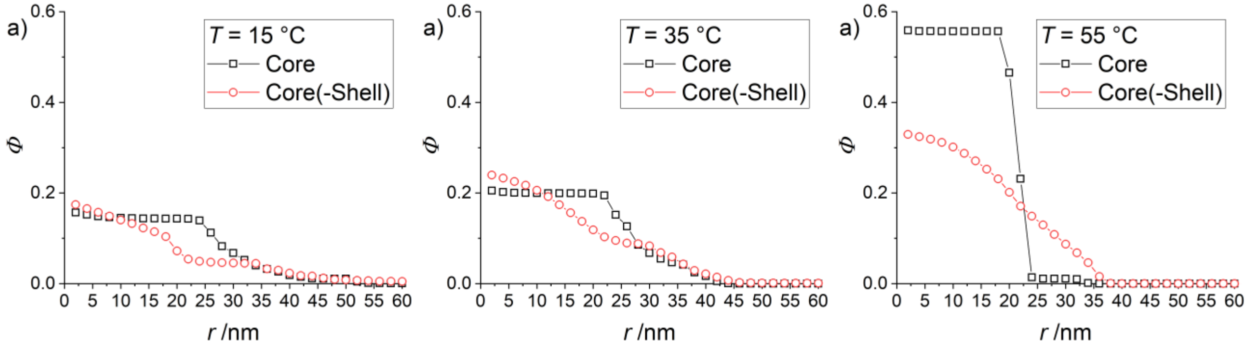

Figure S6: Density profiles of H-pNIPMAM core without shell (black squares) and with a contrast matched D7-pNNPAM Shell (red circles) with a CCC of 10 mol%.

Effect of the B-Spline

In a first approach, we have investigated the necessity of smoothing the density profiles using B-splines (Origin). As shown in the Figure below, this was not needed.

Figure S7 shows the smoothening effect of the B-spline used in Figure 7. The black dashed lines show the not smoothened density profiles, respectively.

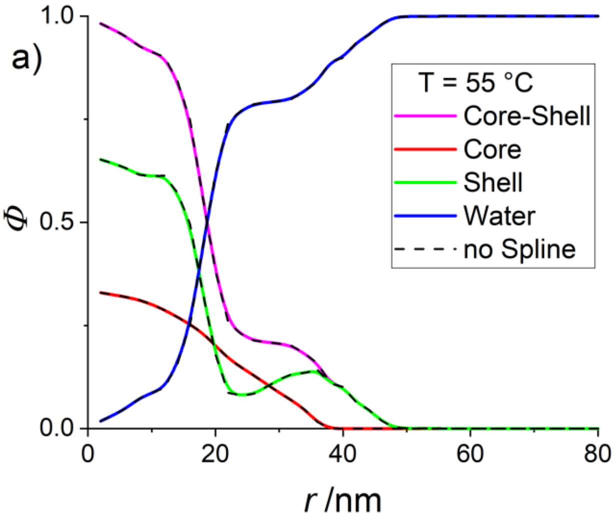

Figure S7: Density profiles of a pNIPMAM-pNNPAM microgel system with ca CCC of 10 mol% at 55 °C with B-spline (colored lines) and the not smoothed data (dashed black lines), respectively.

Angle-dependent PCS

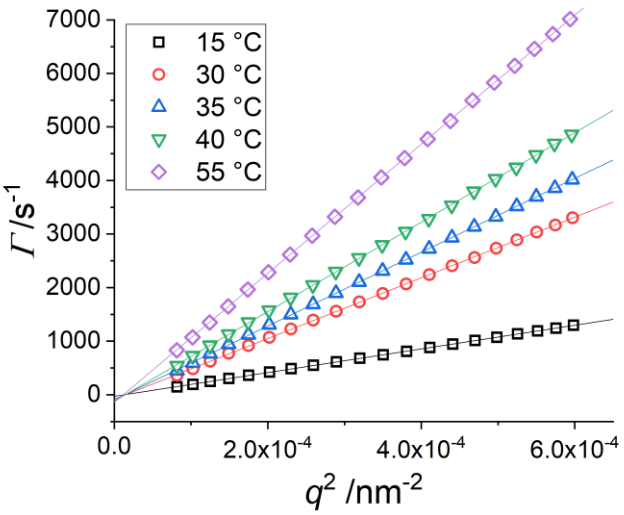

Figure S8: Angle dependent PCS of a H-pNIPMAM core with H-pNNPAM shell and a CCC of 10 mol%.

Sanity Check of the RMC Algorithm

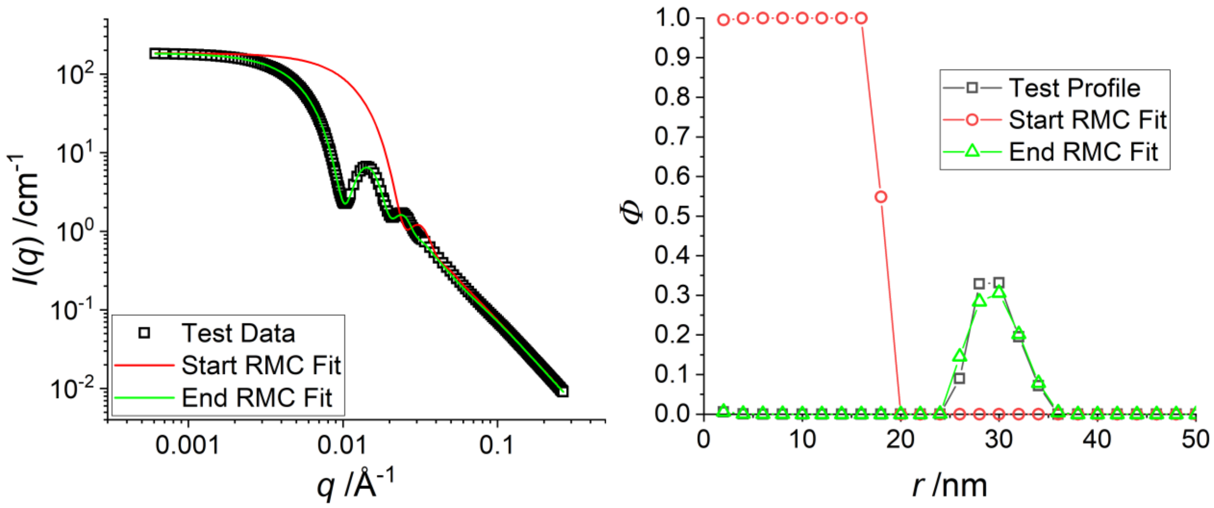

Figure S9: Sanity check of the RMC algorithm. With the tested profile and its intensity curve (black), the density profile at the start of the RMC fit (red) and the density profile at the end of the RMC fit (green). **Left:** Intensity curves. **Right:** density profiles.

Reversibility check

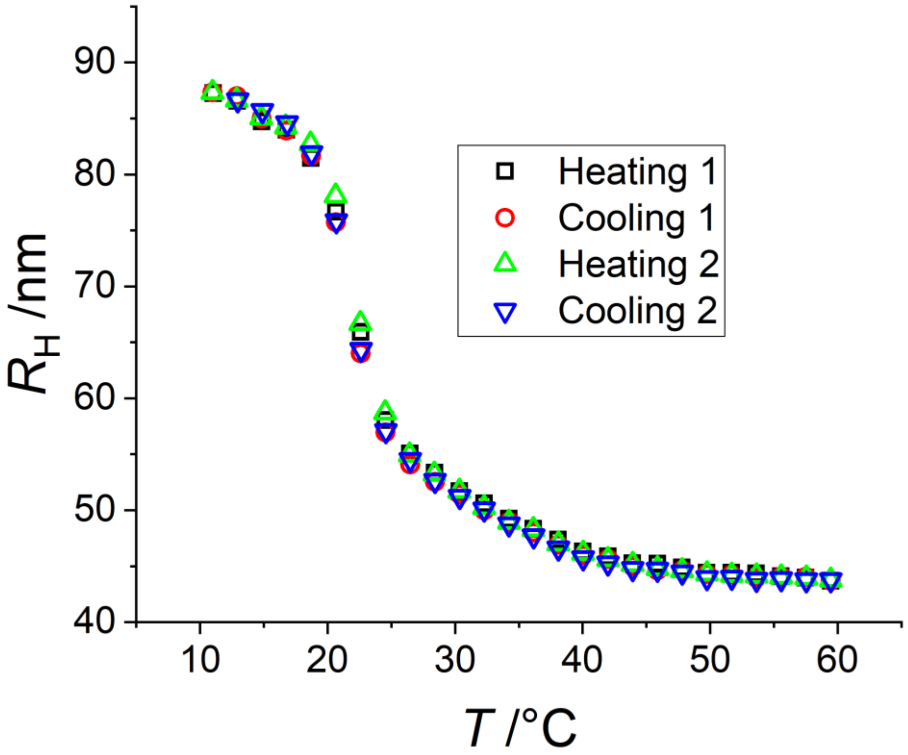

Figure S 10: Hydrodynamic radius as a function of the temperature for a heating-cooling-heating-cooling cycle of a pNIPMAM-pNNPAM core-shell microgel with a CCC of 10 mol%. The measurements were done in H_2O. The equilibration time was 25 minutes after each temperature change. The swelling/de-swelling transition is fully reversible and no hysteresis is observed.

RMC Fit without low *q* data

We applied our RMC algorithm on the same SANS data set with and without the data at very low q where the structure factor $S(q)$ is relevant. Figure S11 shows that the revealed density profile is the same in both cases for 15 and 55 °C. Thus the structure factor does not affect the density profiles.

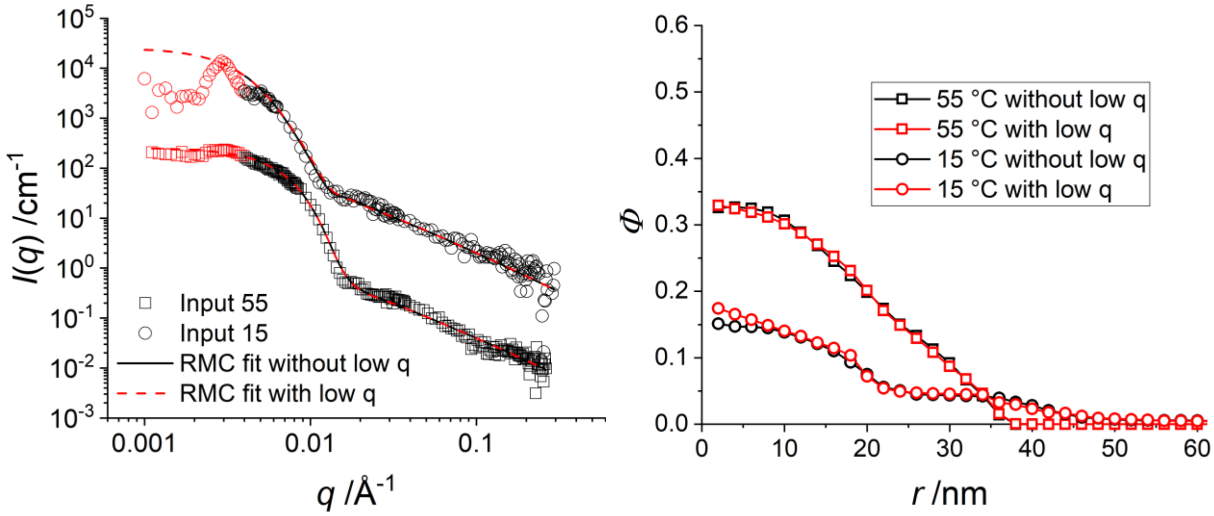

Figure S11: RMC simulations with and without the SANS data at low q where the structure factor is relevant. **Left:** SANS intensity curve with RMC fits. **Right:** density profiles from the RMC fit.

Comparison of the RMC fit with a fuzzy sphere fit

The fuzzy sphere model has been shown by us to be fully compatible with the core data from Cors et al.[1,2] The model, however, is less general than our form-free multi-shell model. Unexpected density profiles like the ones reported here, with a strong presence of shell polymer in the core, cannot be described with a fuzzy sphere model without adaptation, whereas this result is directly provided by our RMC analysis.

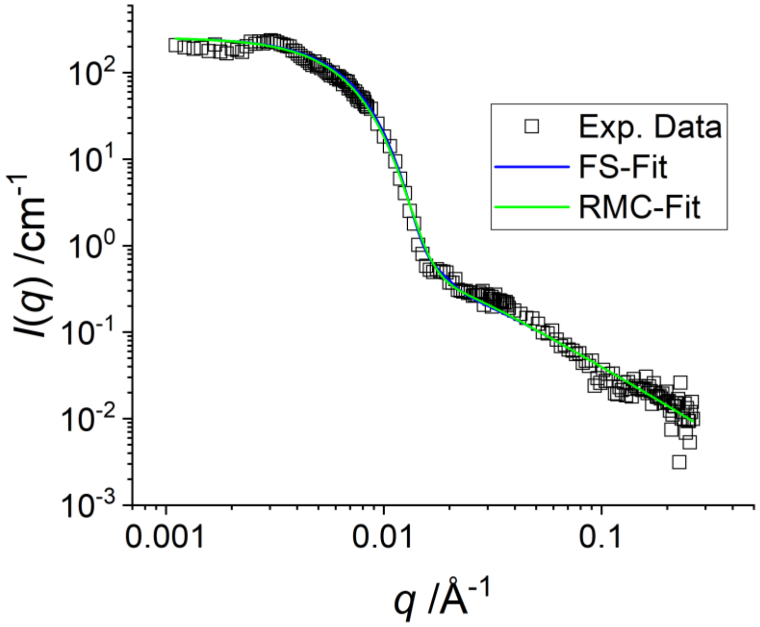

Figure S12: SANS intensity curve of a pNIPMAM-pNNPAM core-shell microgel with a matched shell at 55 °C and a CCC of 10 mol% with a RMC fit and a Fuzzy Sphere fit.

PCS measurement of a pNIPMAM core with an unpolymerized NNPAM "shell"

We performed PCS measurements of a pNIPMAM core and of the same core under the same conditions as the shell synthesis (core, shell monomer, cross linker, surfactant concentration but without the cross linker). The swelling curves are shown in Figure S13.

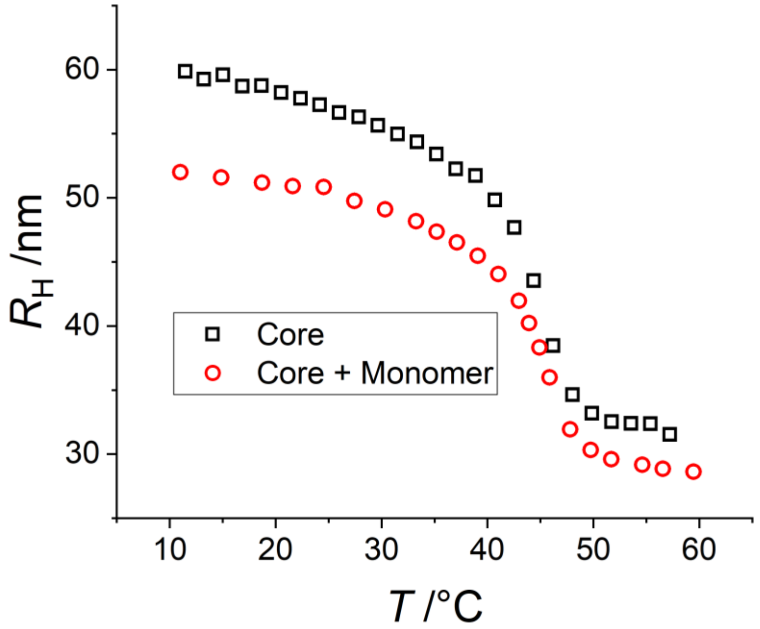

Figure S13: PCS measurement of a pNIPMAM core and the same core under shell synthesis conditions (core, shell monomer, cross linker, surfactant concentration but without the cross linker).

χ^2-values of RMC Fits

The χ^2-values of RMC Fits are listed in the Table below. These χ^2-values are not absolute as they are weighted with the errors in intensity for each *q*-value and are different for each experiment due to merging procedures of different SANS configurations. Altogether, however, they are comparable within one series of experiments, and can be used to compare the quality of the fits.

Table S2: χ^2-values of RMC Fits of the main article.

Figure	Fit	χ^2-values
Figure 2a	55 °C	0.99
	35 °C	0.37
	15 °C	0.13
Figure 3a	15 %	0.87
	10 %	0.99
	5 %	0.94
Figure 4b	55 °C	0.70
	40 °C	0.21
	35 °C	0.30
	30 °C	0.84
	15 °C	0.34
Figure 6a	15 %	0.30
	10 %	0.70
	5 %	0.64

Bibliography

(1) Stieger, M.; Richtering, W.; Pedersen, J. S.; Lindner, P. Small-angle neutron scattering study of structural changes in temperature sensitive microgel colloids. *The Journal of Chemical Physics* **2004**, *120*, 6197–6206.

(2) Cors, M.; Wiehemeier, L.; Hertle, Y.; Feoktystov, A.; Cousin, F.; Hellweg, T.; Oberdisse, J. Determination of Internal Density Profiles of Smart Acrylamide-Based Microgels by Small-Angle Neutron Scattering: A Multishell Reverse Monte Carlo Approach. *Langmuir* **2018**, *34*, 15403–15415.

Figure Legend (13 Figures)

Figure S1: Contrast variation of D7-pNNPAM microgels with a CCC of 10 mol%.

Figure S2: Contrast variation of H-pNNPAM microgels with a CCC of 10 mol%.

Figure S3: Intensity curve and density profile of a H-pNIPMAM core with H-pNNPAM shell with 5 mol% CCC at different temperatures.

Figure S4: Intensity curve and density profile of a H-pNIPMAM core with H-pNNPAM shell with 15 mol% CCC at different temperatures.

Figure S5: Density profiles of H-pNIPMAM cores without and with contrast matched D7-pNNPAM shell at different temperatures.

Figure S6: Density profiles of H-pNIPMAM core without and with a contrast matched D7-pNNPAM Shell

Figure S7: Density profiles of a pNIPMAM-pNNPAM microgel system with ca CCC of 10 mol% at 55 °C with B-spline.

Figure S8: Angle dependent PCS of a H-pNIPMAM core with H-pNNPAM shell.

Figure S9: Sanity check of the RMC algorithm.

Figure S10: Hydrodynamic radius as a function of the temperature for a heating-cooling-heating-cooling cycle of a pNIPMAM-pNNPAM core-shell microgel with a CCC of 10 mol%.

Figure S11: RMC simulations with and without the SANS data at low q where the structure factor is relevant.

Figure S12: SANS intensity curve of a pNIPMAM-pNNPAM core-shell microgel with a matched shell at 55 °C and a CCC of 10 mol% with a RMC fit and a Fuzzy Sphere fit.

Figure S13: PCS measurement of a pNIPMAM core and the same core under shell synthesis conditions.
